# Evaluating the potential of underwater television to contribute to marine litter assessments alongside bottom trawling

**DOI:** 10.1371/journal.pone.0324900

**Published:** 2025-06-27

**Authors:** Katja Norén, Filip Svensson, Max Lindmark

**Affiliations:** Swedish University of Agricultural Sciences, Department of Aquatic Resources, Institute of Marine Research, Uppsala, Sweden; MARE – Marine and Environmental Sciences Centre, PORTUGAL

## Abstract

Marine litter presents a global threat to marine ecosystems, human health, and safety. Therefore, it is important to increase our knowledge about spatiotemporal trends of litter in the environment. Bottom trawl surveys provide a practical method for monitoring seafloor litter on the continental shelf, but can have severe negative impacts on the environment. Here we evaluate the potential of an ongoing underwater television survey (UWTV) to also collect litter density data, and develop model-based indices of litter densities integrating coastal and offshore trawl survey data using geostatistical models. Based on our case study along the Swedish west coast, we find that UWTV in its current format may be limited as an alternative to trawling in areas with relatively low densities. There are also clear spatial trends in litter, with the highest densities in near-shores areas currently only included in the national monitoring program. Our results illustrate the potential of combining data, but also the importance of careful sampling designing for marine litter monitoring.

## Introduction

In the Manila declaration, it was recognized that marine litter poses a worldwide threat not only to marine habitats and species but also to human health and safety [1]. Marine litter, especially plastic litter, is found in increasing numbers around the world [2–5]. It is found in a variety of physiographic settings, but high densities tend to be recorded in coastal areas and in submarine canyons [5–8]. Several pathways have been suggested through which marine macro litter could affect marine organisms such as ingestion, entanglement, toxicity and entrapment [9,10]. Studies demonstrating ingestion of plastic litter by seabirds were already published in the late 1960s [11]. Since then, marine litter has been observed to interact with more than 900 species around the world through ingestion or entanglement [12]. Hence, there is an urgent need to monitor trends and identify spatial hotspots of marine litter [13].

The Marine Strategic Framework Directive (MSFD) was established to achieve or maintain Good Environmental Status in EU marine waters. In the MFSD, marine litter constitutes number ten out of eleven descriptors and thus mandates that marine litter on seafloor should be monitored [14]. The International Council for the Exploration of the Sea (ICES) coordinates several scientific trawl surveys gathering data on commercial fish and invertebrate species. In 2011, it was decided to also record litter on a selection of internationally coordinated scientific trawl surveys. Over time, this procedure has been introduced into several different types of trawl surveys. In Sweden, recording of litter on the seafloor is conducted in two internationally coordinated trawl surveys: 1) the International Bottom Trawl Survey (IBTS) in Skagerrak and Kattegat and 2) the Baltic International Trawl Survey (BITS) in the Baltic Sea. In addition, litter is also registered during the Swedish national Coastal Trawl Survey (CTS), which is conducted along the Swedish west coast and its fjords.

The practice of recording marine litter in trawl surveys has raised concerns due to methodological limitations, and several factors point to litter amounts being systematically underestimated. Firstly, fishing gear catch only an fraction (the “catchability”) of objects it encounters. While the catchability is known for many fish species [15], it is not known for marine litter [16]. Secondly, trawl surveys are primarily conducted far from land and only in areas where it is possible to trawl, i.e., on soft bottoms [17]. To overcome some of these problems, acoustic and electromagnetic methods have been suggested as alternatives. These are beneficial as they are less- or non-destructive, and may be conducted in non-trawlable areas and marine protected areas [13,18].

Underwater video and photography-based monitoring is another increasingly popular class of methods used to classify litter, and estimate their abundance and distribution, especially in areas difficult to trawl [19–21]. To provide fishery-independent information to the stock assessment of (*Nephrops norvegicus*) in Skagerrak and Kattegat, Sweden monitors the density of burrows in muddy sediment using an underwater television survey system (UWTV). The video system is mounted on a benthic sledge that is dragged along the seafloor and video analysis is conducted on land [22]. If the bottom substrate is rugged, i.e., contains large boulders or coral reefs, the sledge may also be used as a drop-camera positioned above the seafloor. An example of a photo using the UWTV is shown in Fig 2. Films from this survey are also used to identify megafauna, and could in theory also be used to monitor marine litter. Large parts of the Skagerrak and Kattegat are covered with the UWTV and some areas are partially overlapping with the IBTS and CTS trawl surveys. This overlap enables a direct comparison of the different methods for detecting litter on the seafloor.

**Fig 1:**
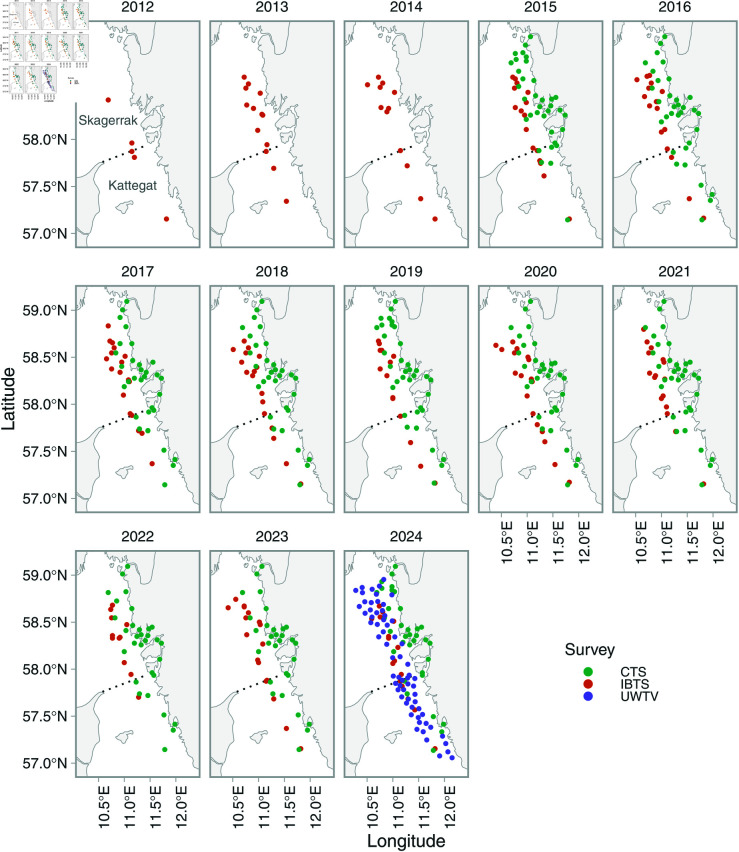
Sampling locations over time. The Coastal Trawl Survey (CTS) is depicted in green, the International Bottom Trawl Survey (IBTS) in orange, and the Underwater TV survey (UWTV) in purple. The IBTS is conducted in Kattegat, Skagerrak and parts of the North Sea but in this study only stations within the area covered by the UWTV survey in 2024 are included (S1 Fig). The dotted line in the topleft panel depicts the Skagerrak/Kattegat border.

The main aim of this study is to evaluate the capacity of the UWTV to detect and quantify litter, as video-based methods are considered more efficient for estimating true litter densities, and have a smaller environmental footprint. The performance of UWTV is assessed through statistical simulation and with data analysis. We also for the first time integrate offshore trawl data with Swedish Coastal Trawl Survey data to acquire model-based indices of relative density, and to quantify spatiotemporal trends in marine litter.

## Materials and methods

### Sampling programs

#### Underwater television survey system (UWTV).

**Fig 2:**
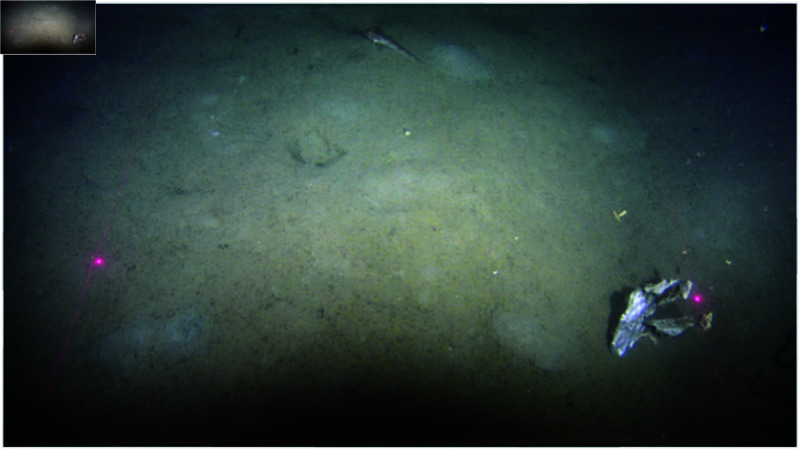
Image from filmed transect. Image of the seafloor with a litter object taken from a transect filmed with an UWTV in 2023 in ICES subarea 4 (Kattegat). The distance between red laser dots is approximately 80 cm. Due to turbidity, it is difficult to say if the object is A2 = plastic sheet or A3 = plastic bag according to the ICES manual [24]. Foto SLU-Aqua, P. Jonsson.

Underwater television Survey System (UWTV) is used to gather data for estimation of the abundance of Nephrops (*Nephrops norvegicus*) [22]. The film from these surveys also used to register benthic macrofauna on the seafloor [23]. In 2024, during the survey of Nephrops grounds in the Skagerrak and Kattegat, the potential of UWTV to evaluate the presence of litter was tested. The survey was conducted during eight days and nights using the Swedish research vessel Svea. In total, 87 UWTV recordings (Fig 1) provided sufficient water clarity and visibility to allow for the identification of megafauna and this task was combined with the identification of marine litter. A typical UWTV-haul runs for 10 minutes at 0.8 knots per hour, thus the area covered in one transect is approximately $148\;{\text{m}}^{2}$. During the analysis, each litter object within a known field of view (0.80–0.85 meters, indicated by laser dots) was registered and the amount of litter per filmed transect is transformed to litter per ${\text{km}}^{2}$ (Fig 2). The registration of litter objects follows the manual produced by the ICES working group for marine litter, WGML [24].

#### International bottom trawl survey (IBTS).

The International Bottom Trawl Survey (IBTS) has been conducted by Sweden in the Skagerrak and Kattegat in the first quarter since the 1980s, and in quarter three since 1991. These surveys are primarily conducted to estimate the number of 0- and 1-year old fish of different commercial species. Surveys and sampling of catch follows the IBTS manual [25]. The fish are caught using a GOV-trawl (Chalut à Grande Ouverture Verticale), which was originally designed to catch herring *Clupea harengus*. The codend of the GOV-trawl features a 20 mm mesh and the width of the trawl (wing spread) varies somewhat with water depth but is generally between 20 and 25 m [25]. Each haul is 30 minutes with a speed of 4 knots, and between 40–50 hauls are made each quarter in the Skagerrak, Kattegat and eastern North Sea combined. In addition to measuring and recording different fish and invertebrate species, litter is also recorded since 2012 following the ICES trawling litter manual [24]. The number of IBTS hauls coinciding with the area covered by UWTV in 2024 varies by year (Fig 1). Only IBTS stations within the area covered by the UWTV in 2024 are included in this analysis. Swedish IBTS data was downloaded from DATRAS (https://www.ices.dk/data/data-portals/Pages/DATRAS.aspx) [26].

#### Coastal trawl survey (CTS).

The Coastal Trawl Survey (CTS) is performed once a year with the purpose of monitoring species composition and recruitment in the benthic fish community in the fjords and along the Swedish west coast [27]. Since 2013, the survey is conducted in the third quarter using a fishing trawl called “FiskeTrål Norden” with a 16 mm mesh in the codend and a width of the trawl (wing spread) between 9–14 m depending on depth. The haul duration is 30 minutes, with a speed of 2.5 knots, and approximately 30 hauls are made each year (Fig 1). In addition to measuring and recording different fish and invertebrate species, litter is also recorded since 2015 following IBTS and BITS manuals and more recently the ICES manual from 2022 specifically regarding marine litter [24,28,29].

### Data analysis

#### Simulation.

**Fig 3:**
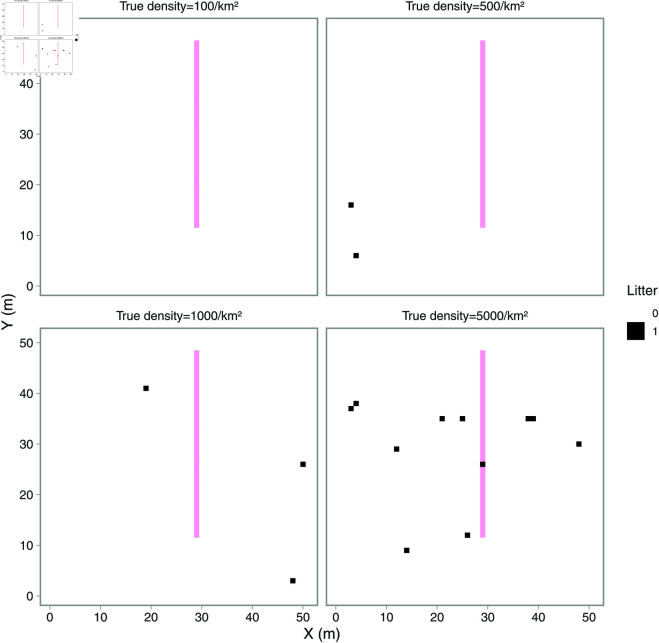
Example of a single replicate of a randomly filled spatial grid. Litter densities are 100, 500, 1000, 5000 items per ${\text{km}}^{2}$. Black grid cells indicate presence of litter. The pink line corresponds to a randomly placed straight UWTV transect. For visualization purposes, we have used relatively high litter densities, zoomed in on a 50×50 m portion of the full grid, and divided the transect by 4 (hence, in the simulation experiment, the UWTV transect would be 4 times as long).

We used simulation testing to evaluate the performance of the UWTV to sample marine litter. The approach consists of the following steps:

1. Generate a 1000×1000 m spatial grid.
2. For each litter density scenario, randomly distribute litter objects over the grid to get values for presence or absence of litter for each ${\text{m}}^{2}$. Only one litter object is allowed per ${\text{m}}^{2}$. A hypothetical smaller grid is shown as an example in Fig 3.
3. For each replicate and litter density scenario, apply a random sample representing the UWTV-method. A single random sample is made up of 148 consecutive cells distributed horizontally or vertically over the grid (start location determined randomly), each cell is $1\;{\text{m}}^{2}$ and this is intended to mimic a UWTV transect which on average is $148\;{\text{m}}^{2}$.
4. Repeat step 3 for each sample size scenario (we chose 50, 100, 200, 500, 1000 transects, each with a size of $148{\text{m}}^{2}$). These sample size scenarios are intended to both include relevant sample sizes (in this study 87 transects were filmed) and also more unrealistic examples such as 1000 transects. For each litter density and each sample size scenario transects were distributed 1000 times.

From the simulation experiment, we calculated: (1) the proportion of replicates (across the 1000 replicates) with empty 0 litter recorded during UWTV transects, for each litter density scenario and each sample size, (2) the mean average litter density across replicates, by litter density and sample size. R functions for the simulation experiment were developed partly using the large language model Claude [30].

#### Statistical modelling.

To estimate annual trends in relative litter abundance, we used geostatistical generalized linear mixed models (GLMMs), similar to those used in species distribution modelling. Litter objects are categorized into 42 litter categories that cover a range of materials and specific groups of objects, such as “plastic bottle” or “metal can”. In this study, we model the total abundance density of all litter categories per haul. Since litter density data contain zeroes and positive continuous observations, we used a delta (hurdle) model, with a binomial and a Gamma component. This was fit as a so called “Poisson-link” delta model, which has the flexibility of a classic delta model [31], but avoids the assumption that the two components are statistically independent [32]. To account for spatial structure in the data, we included spatial random effects in the form of Gaussian Markov random fields (GMRFs) using the SPDE approach [33]. The full model for a given component (binomial or Gamma) can be written as:

$$𝔼[y_{s,t}]=\mu_{s,t},$$ (1)

$$\mu_{s,t}=f^{-1}\left(X_{s,t}\beta \right),$$ (2)

$$\omega_{s}\sim MVN(0,\Sigma_{\omega }),$$ (3)

$$\delta_{t=1}\sim \mathrm{MVNormal}(0,\Sigma_{\epsilon }),$$ (4)

$$\delta_{t>1}=\rho \delta_{t-1}+\sqrt{1-\rho^{2}}\epsilon_{t},\epsilon_{t}\sim \mathrm{MVNormal}\left(0,\Sigma_{\epsilon }\right),$$ (5)

where $y_{s,t}$ is the response variable (number of litter items per ${\text{km}}^{2}$) in location s at time *t*, μ is the mean, ${\text{f}}^{-1}$ is the inverse link function, X is the design matrix for fixed effects with corresponding coefficients β. We included a categorical effect of survey to account for different catchability of the gear used in the two surveys CTS and IBTS. This because there is a difference in the average densities between the surveys, and we want to test if this is due to gear or sampling area (S1 Fig and S2 Fig). We also added independent intercepts for each year by including year as a factor variable, following common practices in fish stock index standardization [34,35]. This corresponds to the assumption that marine litter is being replaced and added every year. Our initial aim was to include also the UWTV data in this model. However that was not possible since no litter was detected in 2024 (see Sect Results).

Since we do not know which processes and variables give rise to spatial patterns in litter data, we rely on latent variables to model spatial patterns in the data. These are included as spatial and spatiotemporal random effects ($\omega_{s}$ and $\epsilon_{s}$, respectively), assumed drawn from Gaussian Markov random fields (GMRFs) with covariance matrices $\Sigma_{\omega }$ and $\Sigma_{\epsilon }$ constrained by anisotropic Matérn covariances function [36]. Spatial random effects correspond to spatially structured latent variables that are constant over time (e.g., currents, depth, bathymetric slope). Spatiotemporal random effects are also spatial latent variables, but with a separate field for each year. These therefore represent un-observed processes that cause spatial patterns in the data that are allowed to vary from year to year (e.g., weather). Anisotropy means the spatial correlations can depend on direction. This is fitting in our case study since we are modelling coastal data and spatial patterns likely change more going from near shore to offshore than up and down the coast (S4 Fig). Initial exploration revealed strong correlation between subsequent spatiotemporal random fields. Hence we opted to model these fields as AR1 (first-order autoregressive), where ρ is the correlation coefficient between subsequent spatiotemporal random fields. The correlation structure between spatiotemporel random field also helps informing predictions in years when samples were scarce in place (e.g., 2012 in Fig 1), compared to if we had modelled them as independent each year and there would be no process in the model besides the constant spatial random field that could inform the density in that location. The Stochastic Partial Differential Equation (SPDE) approach [33] requires piece-wise linear basis functions defined by a triangulated mesh. We defined this mesh using triangles with a cutoff distance (minimum distance between vertices) of 3 km and kept all other arguments in the R-function fm_rcdt_2d_inla() in the package fmesher [37] at their defaults (S4 Fig).

Based on exploratory data analysis, we consider three alternative models: (1) only spatial random effects (2) only spatiotemporal random effects, and (3) spatial random effects for the binomial model and spatiotemporal random effects for the Gamma model. We use marginal AIC to select the more parsimonious model.

To evaluate trends in average litter densities, we made conditional predictions for each independent year. Next, fit a model to the annual estimates, using the inverse of the CV (coefficient of variation) for each year as weights to incorporate the varying uncertainty in the annual estimates.

We fit the models using the R (version 4.3.2) [38] package sdmTMB [39] (version 0.6.0.9015). The sdmTMB package uses automatic differentiation and the Laplace approximation from the R package TMB [40], along with sparse matrix structures constructed with the SPDE method [33] using the R package fmesher [37]. Parameter estimation was performed via maximum marginal likelihood using the nlminb [38] non-linear minimizer. We ensured the models converged by verifying that the Hessian matrix was positive definite, that the maximum absolute log-likelihood gradient for the fixed effects was less than 0.001, and that no random field marginal standard deviation was larger than 0.01. To ensure that the model was consistent with the observed data we visually inspected simulated quantile residuals [41,42], calculated using the R package DHARMa [43] (S3 Fig). All map base layers are in the public domain. We use the R package rnaturalearth [44] to produce these.

## Results

**Fig 4:**
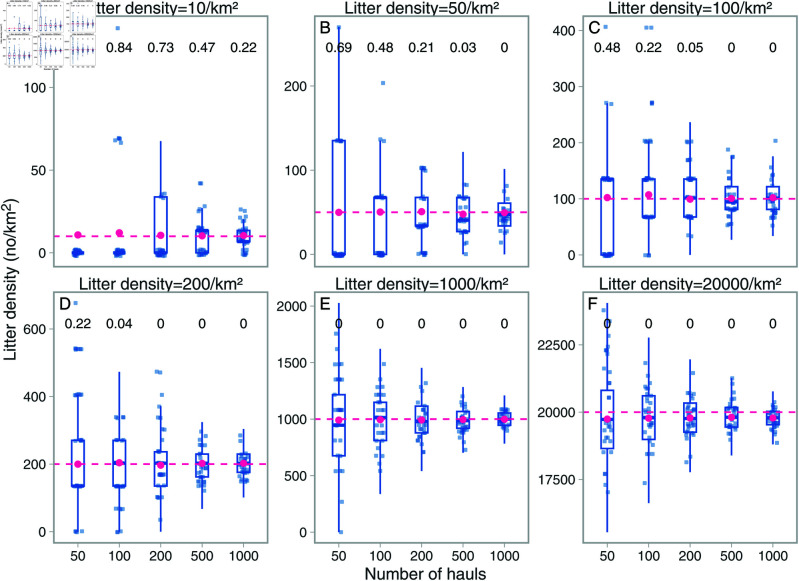
Results from the simulation experiment. Each panel (A–F) corresponds to a litter density scenario, and each blue point represents the estimated mean density for that sample size (number of hauls) (x-axis) and iteration. To avoid overplotting, we randomly sampled 30 of the 1000 blue points and added a small horizontal and vertical jitter. The pink circles correspond to the mean litter density across all 1000 replicates. The horizontal pink line depicts the true litter density in the simulation (also indicated in the panel title). The number on the top corresponds to the proportion of the 1000 simulations that did not catch a single litter item in that sample size scenario.

In the simulation experiment we found that across 1000 replicates for each combination of litter density and sample size, the UWTV with its current sampling size and area swept is inadequate to sample litter at these *relatively* low densities. For example, when the density was 10 items per ${\text{km}}^{2}$, the percentage of replicates of the experiment where the survey did not capture a single litter item was as high as 92% when the sample size was 50, and 84% when the sample size was 100 (the number of hauls in the 2024 UWTV survey was 87) (Fig 4A). Moreover, while the overall mean across all 1000 replicates was close to the true mean (pink points in Fig 4A), individual replicates either estimated 0 litter density or severely overestimated the true mean by a factor of >10 in some cases. That was because if a litter item was recorded (a 10% probability), the density will be very high given the small area sampled. Similarly, when the true litter density was 50 (Fig 4B) and the sample size was 100, single replicates estimate litter densities range from 0 to ≈250 per ${\text{km}}^{2}$, where the higher value was an overestimation by a factor 5. The simulation experiment shows that with litter densities of 50 (comparable to the trawl surveys), it would require a minimum of 500 hauls to have a 97% probability of observing a minimum of at least a single litter item across 1000 iterations (Fig 4C). At higher litter densities, the number of hauls needed to have similar values was lower. At litter densities of 1000 per ${\text{km}}^{2}$, all replicates find litter.

**Fig 5:**
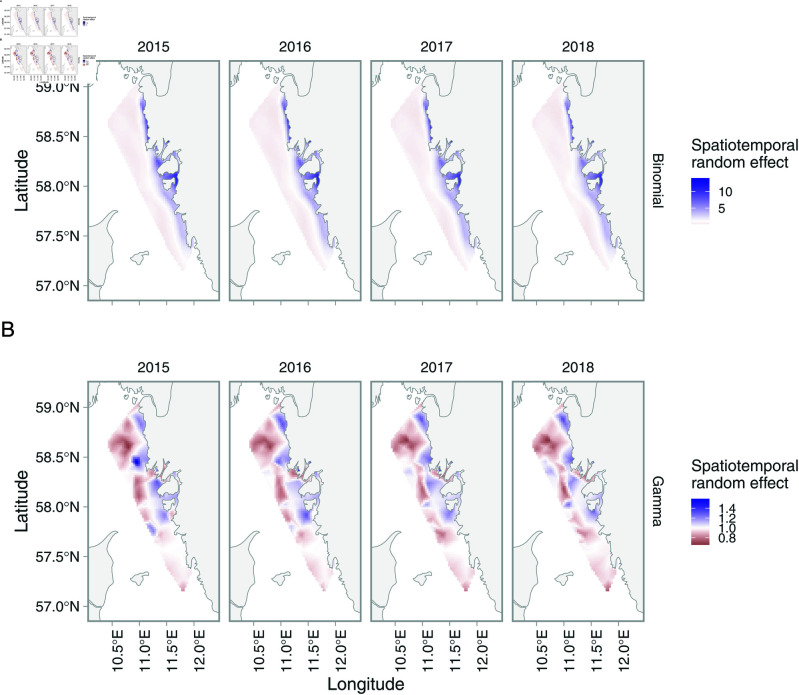
Spatiotemporal random effects for the binomial model (top row) and the Gamma model (bottom row) for selected years (2015–2018).

From the spatiotemporal models fitted to trawl survey data, we found that the marginal Akaike information criterion (AIC) supported the model where both components had the same random effect structure (spatiotemporal random effects for both the binomial and Gamma components). However, the model with a spatial random field for the binomial model and a spatiotemporal field for the Gamma model was nearly indistinguishable in terms of marginal AIC (S1 Table). This is also evident in that the spatiotemporal random fields are more similar from year to year in the binomial model than for the Gamma model (Fig 5). The correlation between consecutive spatiotemporal random fields (ρ) was very high (0.99) in the binomial model, and relatively high in the Gamma model (0.75).

The random effects in addition showed a clear directionality in the spatial correlation, meaning the range where correlation effectively disappears was longer going along the coast (northwest to southeast) than from coastal to offshore (Fig 5 and S4 Fig). This means that locations are more alike each other in the north-east direction than in the west-east direction over short distances. This distance was larger for the binomial model, which illustrates that the presence of litter largely depends on the distance to the coast. There was no clear statistical difference between the survey intercepts, meaning the differences in mean catch was due to the Coastal Trawl Survey (CTS) sampling in higher density areas, rather than it having higher catchability (S1 Fig and S2 Fig).

**Fig 6:**
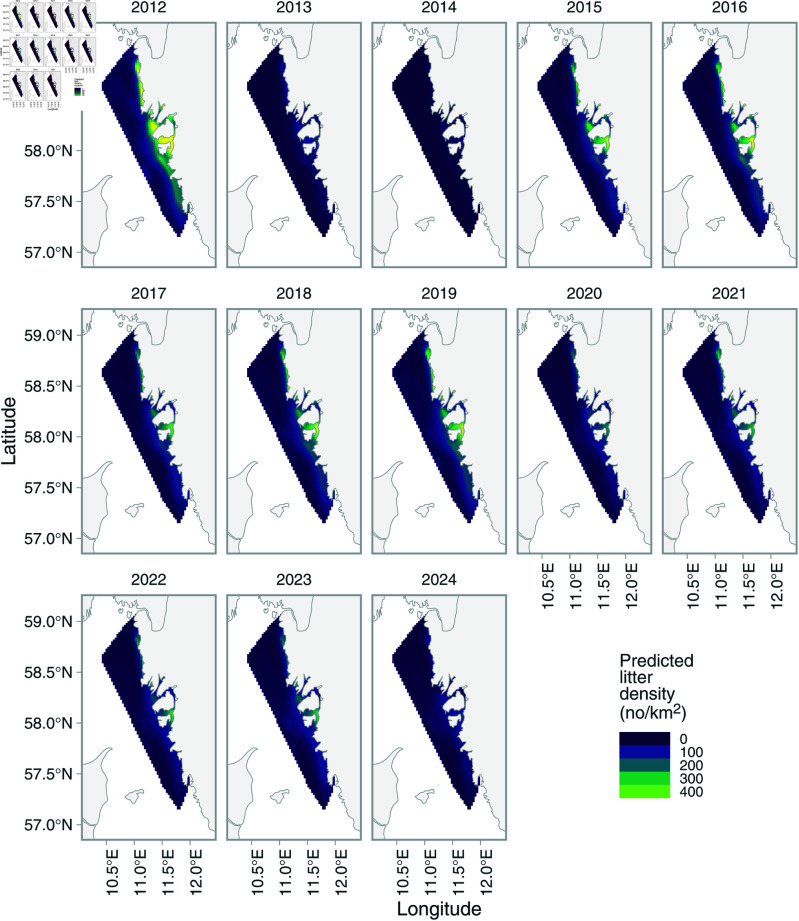
Predicted litter densities from the spatiotemporal model for the years 2012–2024. To better visualize the spatial patterns, values greater than the 99% quantile (479 items per ${\text{km}}_{2}$) are set to the highest color.

**Fig 7:**
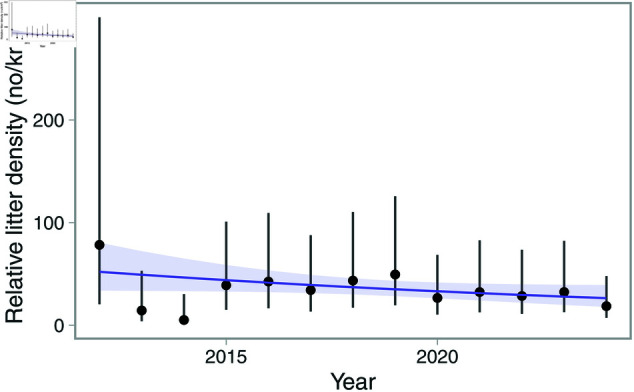
Conditional predictions of litter density for the IBTS level (points) and 95% confidence interval (vertical lines) without random effects. The purple line illustrates trends in annual estimates of mean litter densities and is the prediction from a GAM year modelled as a penalized spline and the inverse of the CV for annual predictions as weights.

The same spatial pattern was also evident in the combined model predictions (Fig 6). The combined predictions also revealed fluctuations over time, with the highest densities in the first year of the time series (Fig 7). Conditional predictions for year omitting the random effects with the model showed that average litter densities ranged between 5 [95% CI: 0.86–30.3]–78 [95% CI: 20.4–300] items per ${\text{km}}^{2}$, with a mean of 34 across all years. The linear effect of year from the weighted regression on annual litter densities was negative (a decline in density by -2.09 per year), but the confidence interval of the slope overlapped 0 [95% CI: –4.17–0.0039] (Fig 7).

**Fig 8:**
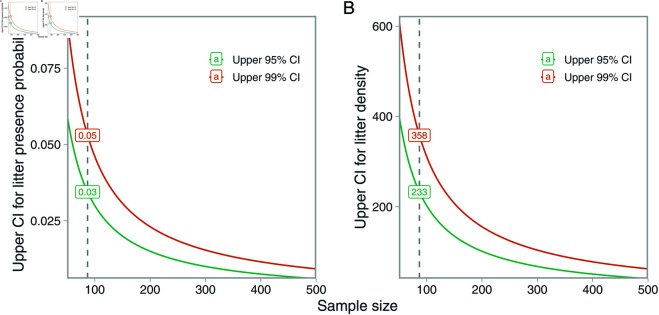
Effect of sample size on upper confidence intervals of probability of detection. Illustration of how the upper confidence interval (95% in green and 99% in orange) for the probability of litter being present in a given haul (A) and the estimated density that corresponds to (B) change as a function of sample size if no hauls record any litter, using the “rule of three” and a sampled area of $148\;{\text{m}}^{2}$. The lower confidence interval is always zero.

The UWTV survey did not record a single litter item in the 87 UWTV transects that were conducted in 2024. While we do not know the true litter density in the area sampled by the UWTV, the simulation, though being a simplification of reality, does indicate that under probable densities (approximately 100 items per ${\text{km}}^{2}$), there is a 22% chance of that no litter are observed in 100 transects (Fig 4C). When no successes (no litter) are observed in a series of binomial trials, one can estimate the confidence interval of probabilities of occurrence using the “rule of three” [45,46]. The rule of three is a simple method for sample sizes larger than 30 that can be used to estimate the upper confidence interval for the probability of presence by 3/*n* (99% confidence interval is given by 4.61/*n*) [45], where *n* is the number of trials (transects in this case). With *n* = 87, we found that the 95% confidence interval for probability of presence of litter in a given transect was between 0 and 0.034 (or 0 and 0.053 for the 99% confidence interval) (Fig 8A). Moreover, when a litter object is recorded by the UWTV, the estimated density will be extremely high in that specific transect (as we showed also in the simulation study), because the swept area is small. In 87 transects, the expectation for the upper 95% confidence interval for the number of transects with litter is 0.034 × 87≈3. The average upper 95% confidence interval of litter density across those 87 transects is 233 items per ${\text{km}}^{2}$ (84 transects recording 0 density and three transects recording a density of 6757 items per ${\text{km}}^{2}$ [1/(6757/1000000)]) (Fig 8B). However, this is a simplification, because the UWTV could in reality record more than one litter item per transect. To further provide insight into how the confidence interval behaves under different scenarios where few transects contain litter, we calculated confidence intervals for varying number of transects with litter and varying sampling sizes using the Agresti-Coull method [47], implemented in the R package DescTools [48] (S5 Fig).

## Discussion

In this study, we used data and simulation experiments to determine the ability of Underwater TV (UWTV) to replace the more destructive trawl survey methodology for collecting data. We then applied geostatistical models to the trawl data to determine levels, trends, and spatiotemporal patterns in marine litter. We conclude that the UWTV sampling is not suitable for contributing to monitoring of marine litter in its current form. This is because it did not record any litter, likely due to the UWTV’s relatively small “swept area” compared to a trawl, combined with its use in offshore areas where our spatiotemporal models showed lower litter densities compared to coastal regions. While we can still calculate upper confidence intervals for probability of occurrence, we cannot provide any expected values of litter densities, which is the aim of the survey and needed for monitoring trends in estimated litter densities. Current trawl surveys also provide large amounts of data on different categories of litter found on the seafloor. With zero or few findings in the current UWTV setup this information is lost. Important to emphasize is also that the current UWTV setup has a lower geographical coverage of Skagerrak, Kattegat and the North Sea compared to the IBTS trawl survey.

Our model based on two surveys, showing similar results in the overlapping area, provides strong evidence that litter densities are higher closer to shore. This is in line with other studies, for instance in the Saronikos Gulf in Greece [17]. This calls for an expansion of the UWTV survey towards coastal areas if one believes that filming the seafloor is better to get a true estimate of amounts of litter (recall it does not have the same issues with catchability as a trawl haul and has a lower environmental footprint). Preferably the UWTV should be conducted in regions that have not been previously sampled in the CTS as there is a risk that yearly trawling along the same transects have removed litter.

In the future, trends in marine litter will likely to a higher degree stem from estimates integrating multiple data sources [17]. In that case, a model similar to the one used here could be used to integrate those different datasets, which is one of the strengths of model-based trends [49]. Using multiple data sources, that complement each other (e.g., in terms of location of sampling) can increase accuracy and reduce uncertainty in annual indices [50]. It could also help expand the survey domain into areas that are no-trawleable [17]. In a similar application aimed to estimate rockfish and flatfish densities from towed cameras and bottom trawls, [51], spatial differences in the catchability between camera and trawl were found. Such analysis could be applied also in our case study, because while it is hypothesized that UWTV has a higher catchability/detectability, it remains to be tested with a model that integrates both data sources.

Prior to developing joint models integrating multiple data sources, it is important to further evaluate UWTV monitoring in our case study. Specifically, the possibility to increase transect length, number of deployments, or survey length, or any other change that increases the probably of detecting litter in a given deployment should be explored. A typical tow in our case study is $148\;{\text{m}}^{2}$, which seems to be on the lower end compared to similar studies. For example, the average area covered by the ROV transects in [17] was $3540\;{\text{m}}^{2}$. This is a 24 times larger area covered, and in an area with litter densities as high as 251250 items per ${\text{km}}^{2}$ [52]. These densities are more than 10 times higher than the highest density we considered in the simulation study, and 5000 times higher densities than what we find on average in our domain (Fig 7). This comparison illustrates clearly the importance of survey design that takes into account local conditions.

The spatiotemporal model used here is largely inspired by species distribution models and models used to create model-based indices of abundance in fisheries science [53]. However, there are some interesting differences. The spatial distribution of species results from the interplay between environmental and ecological processes (competition, predation) [54,55]. For instance, the strong association species may have to certain environmental variables (e.g., depth or temperatures) can be used to improve the underlying spatiotemporal model and thereby indices of relative abundance [49,53]. In contrast, unlike biological organisms, the drivers of the litter abundance and distribution are likely more elusive, or they may at least vary across locations. Which of these drivers are most influential are largely unknown and likely depend on the material of the litter, where plastics may be more easily transported with currents while more dense litter or larger object are not removed easily [56,57]. There could also be areas acting as sinks, e.g., shelfs and deep sea areas [58]. Hence, it is difficult to *a priori* know which covariates to include in a model, and more research on this is needed to improve models. One such example is [59], in which high litter densities where associated with high vessel traffic intensity and strong bottom current flows. Such findings could help improve model fit. In this study, we instead of covariates used an approach based on Gaussian Markow random fields. In similar applications [60,61], researchers have used similar models with smoothers of latitude and longitude, and different options for modelling the temporal trends (linear, smooth, independent means). Overall these are similar models, but a benefit of using our approach is that it can determine the range at which spatial correlation disappears (and the directionality of it). While we have only applied this to a case study on the Swedish west coast, we believe it could be applied in general for estimating marine litter levels.

## Conclusion

This study highlights an important potential challenge when integrating or replacing trawling methodology with underwater television. The significant difference in area coverage between these methodologies might be an overlooked factor. In the international trawl-surveys in this study, one IBTS haul covers approximately 468 times the area of one UWTV haul. We present several scenarios that we hope can be of practical guidance for developing monitoring programs, based on results from simulation experiments and spatiotemporal trends in litter based on trawl data. While we can estimate upper confidence intervals of litter density, the probability of litter detection per UWTV transect must increase for providing point estimates of litter densities. To be of practical use for management, both point estimates and uncertainty interval are likely required. To achieve higher detection rates, transect length and the number of transects performed need to be adjusted. For example, our simulation reveals that with 50 hauls in an area with 10 items per ${\text{km}}^{2}$, no litter was found in 920 out of 1000 iterations of the simulation experiment. Only at densities of 1000 items per ${\text{km}}^{2}$ did each of the 1000 simulations with 50 hauls detect at least one litter object. One could argue that a correctly adjusted sample size should have a high probability of detecting at least one litter item. With a higher sampling intensity, lower densities can be detected. Alternatively, as our models indicate, litter densities are higher near the coast, and there may be many benefits of relocating UWTV effort to these areas. The higher densities means the effort may not have to increase dramatically to achieve a higher detection rate, and the lower impact of UWTV compared to trawl is likely more suitable for sensitive coastal habitats. In summary, this study demonstrates how geostatistical models can be used to combine data sources, estimate standardized indices of litter density, and identify spatiotemporal hotspots in litter. Such information is extremely valuable for designing monitoring programs and where to focus resources.

## Supporting information

S1 Fig Trends in mean litter and location of samples. Mean litter densities (A) by survey (green = CTS, orange = IBTS), over time, and location of samples (B) with polygons depicting concave hulls of the survey extent. Note the CTS is split in two, where CTS in the UW/IBTS polygon is denoted CTS offshore (triangles) and coastal data are denoted CTS coastal (points), to illustrate that the differences in mean litter between CTS and IBTS is due to spatial differences in litter density and sampling area (see also <s002>S2 Fig and Fig 5).
(PDF)

S2 Fig Effect of survey on litter density from the spatiotemporal model. (PDF)

S3 Fig QQ plots. QQ-plots based on simulated quantile residuals for the combined predictions of the litter density models where fixed effects are held at their maximum likelihood estimate and random effects taken from a single approximate posterior sample.
(PDF)

S4 Fig SPDE meshes and spatial range. Panel A depicts the SPDE mesh for the litter model, and in panel B, the ellipses depict the spatiotemporal range (the distance at which correlation is effectively independent) for the two model components (green = binomial, orange = Gamma).
(PDF)

S5 FigRelationship between litter density and sample size.Litter density estimates and 95% CI for varying sample sizes (number of hauls) and number of hauls with litter per sample size using the Agresti-Coull method (note a haul with litter can only contain one litter object in this hypothetical example). Haul area: $0.000148\;{\text{km}}^{2}$. The solid line depicts the mean and the ribbon covers the 95% confidence interval.(PDF)

S1 TableAIC for litter density models.AIC and ΔAIC (AIC for the model relative to the model with the lowest AIC) for all spatial and spatiotemporal GLLMs fitted to litter density data. In model 1, we use a spatial random field for the binomial and Gamma components of the delta-model, in model 2, we replace the spatial random field with a spatiotemporal AR1 random field, and in model 3 we use a spatial random field for the binomial model and a spatiotemporal AR1 random field for the Gamma model.(PDF)
