# Supplementary material for: Evaluating the potential of underwater television to contribute to marine litter assessments alongside bottom trawling
Source: PLoS One. 2025 Jun 27;20(6):e0324900. doi: 10.1371/journal.pone.0324900 (PMC12204539; doi:10.1371/journal.pone.0324900)
Supplement: S1 Fig — Mean litter densities (A) by survey (green = CTS, orange = IBTS), over time, and location of samples (B) with polygons depicting concave hulls of the survey extent. Note the CTS is split in two, where CTS in the UW/IBTS polygon is denoted CTS offshore (triangles) and coastal data are denoted CTS coastal (points), to illustrate that the differences in mean litter between CTS and IBTS is due to spatial differences in litter density and sampling area (see also S2 Fig and Fig 5). (PDF) [file pone.0324900.s001.pdf]

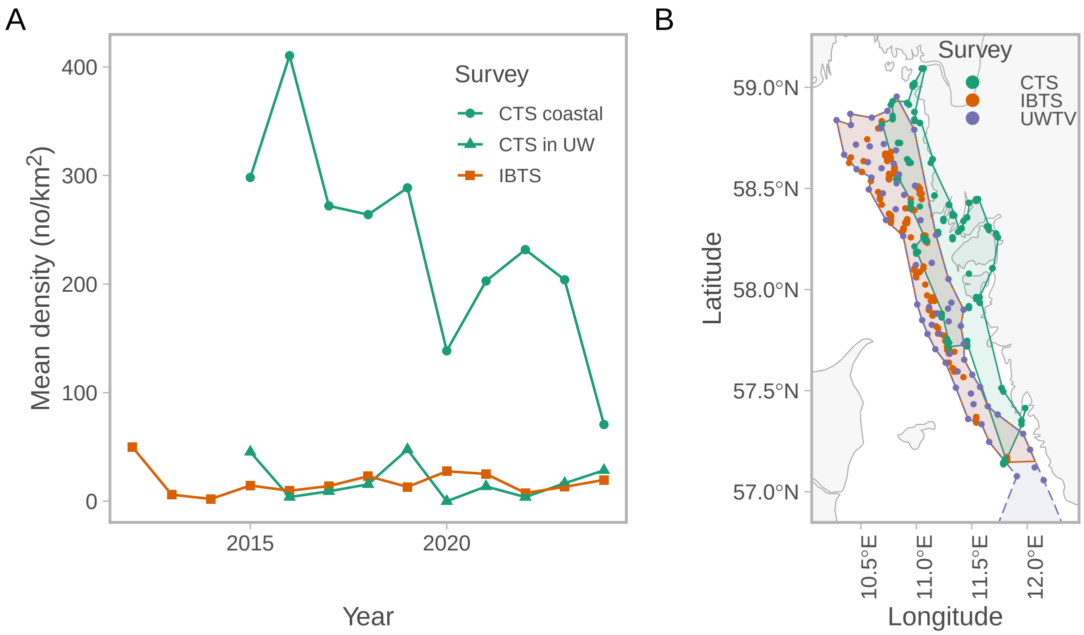

Figure S1: Mean litter densities (A) by survey (green = CTS, orange = IBTS), over time, and location of samples (B) with polygons depicting concave hulls of the survey extent. Note the CTS is split in two, where CTS in the UW/IBTS polygon is denoted CTS offshore (triangles) and coastal data are denoted CTS coastal (points), to illustrate that the differences in mean litter between CTS and IBTS is due to spatial differences in litter density and sampling area (see also Fig S2 and Fig ??).
