# Supplementary figures and images for: Evaluating the potential of underwater television to contribute to marine litter assessments alongside bottom trawling
Source: PLoS One. 2025 Jun 27;20(6):e0324900. doi: 10.1371/journal.pone.0324900 (PMC12204539; doi:10.1371/journal.pone.0324900)

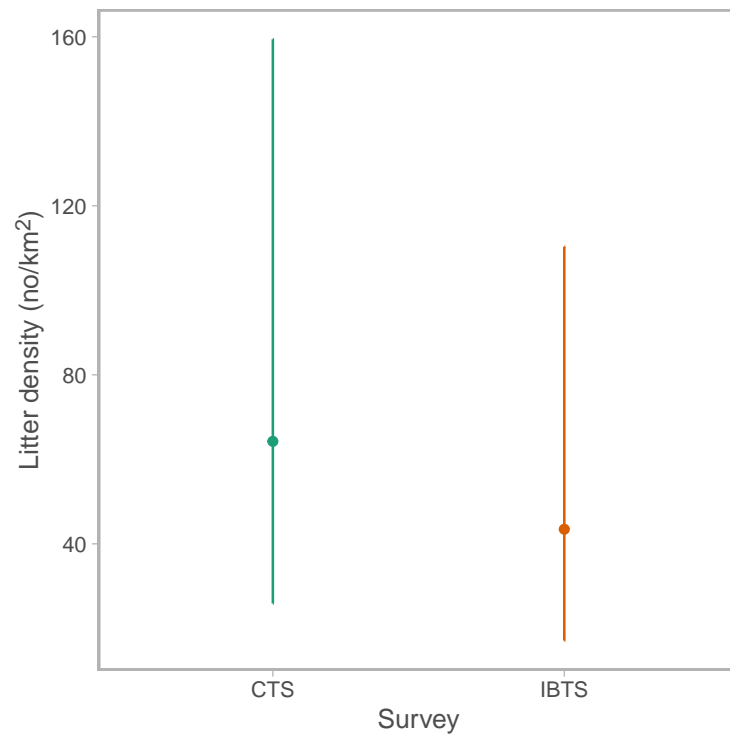

Figure S2: Effect of survey on litter density from the spatiotemporal model.

Supplement: S2 Fig — (PDF) [file pone.0324900.s002.pdf]
