# Supplementary material for: Evaluating the potential of underwater television to contribute to marine litter assessments alongside bottom trawling
Source: PLoS One. 2025 Jun 27;20(6):e0324900. doi: 10.1371/journal.pone.0324900 (PMC12204539; doi:10.1371/journal.pone.0324900)
Supplement: S3 Fig — QQ-plots based on simulated quantile residuals for the combined predictions of the litter density models where fixed effects are held at their maximum likelihood estimate and random effects taken from a single approximate posterior sample. (PDF) [file pone.0324900.s003.pdf]

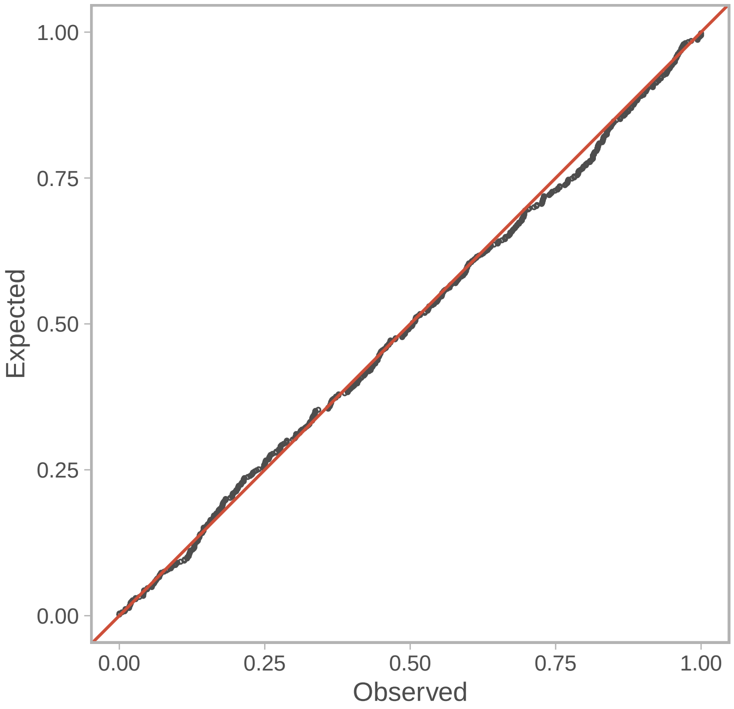

Figure S3: QQ-plots based on simulated quantile residuals for the combined predictions of the litter density models where fixed effects are held at their maximum likelihood estimate and random effects taken from a single approximate posterior sample.
