# Supplementary material for: Evaluating the potential of underwater television to contribute to marine litter assessments alongside bottom trawling
Source: PLoS One. 2025 Jun 27;20(6):e0324900. doi: 10.1371/journal.pone.0324900 (PMC12204539; doi:10.1371/journal.pone.0324900)
Supplement: S4 Fig — Panel A depicts the SPDE mesh for the litter model, and in panel B, the ellipses depict the spatiotemporal range (the distance at which correlation is effectively independent) for the two model components (green = binomial, orange = Gamma). (PDF) [file pone.0324900.s004.pdf]

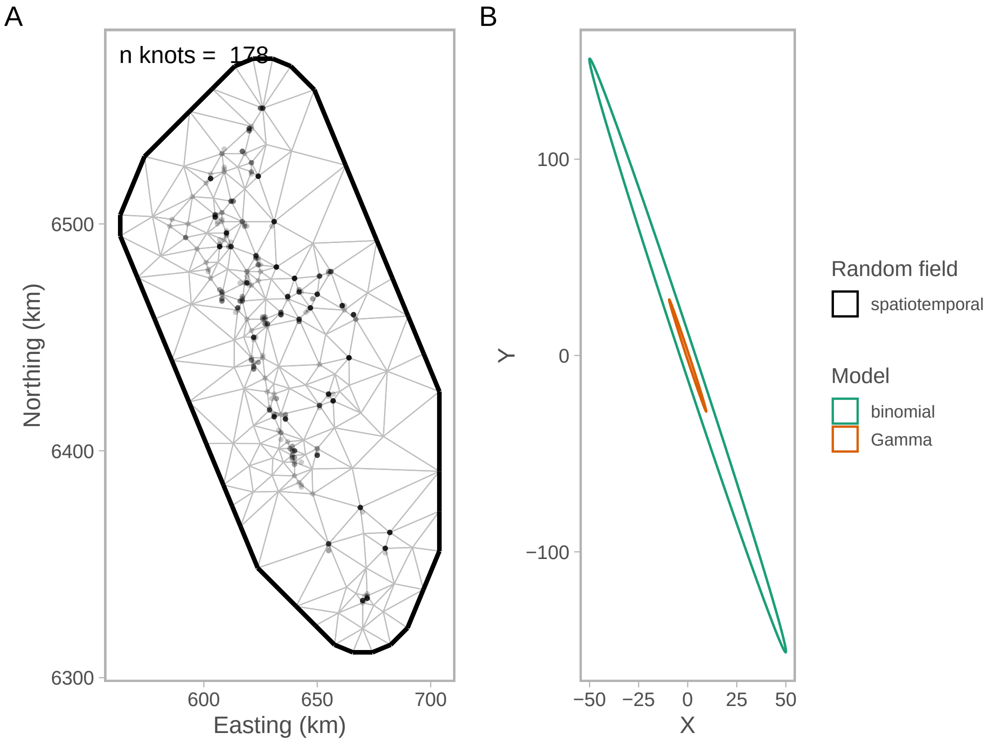


Figure S4: Panel A depicts the SPDE mesh for the litter model, and in panel B, the ellipses depict the spatiotemporal range (the distance at which correlation is effectively independent) for the two model components (green = binomial, orange = Gamma).
