# Supplementary material for: Evaluating the potential of underwater television to contribute to marine litter assessments alongside bottom trawling
Source: PLoS One. 2025 Jun 27;20(6):e0324900. doi: 10.1371/journal.pone.0324900 (PMC12204539; doi:10.1371/journal.pone.0324900)
Supplement: S5 Fig — Litter density estimates and 95% CI for varying sample sizes (number of hauls) and number of hauls with litter per sample size using the Agresti-Coull method (note a haul with litter can only contain one litter object in this hypothetical example). Haul area: 0.000148 km2. The solid line depicts the mean and the ribbon covers the 95% confidence interval. (PDF) [file pone.0324900.s005.pdf]

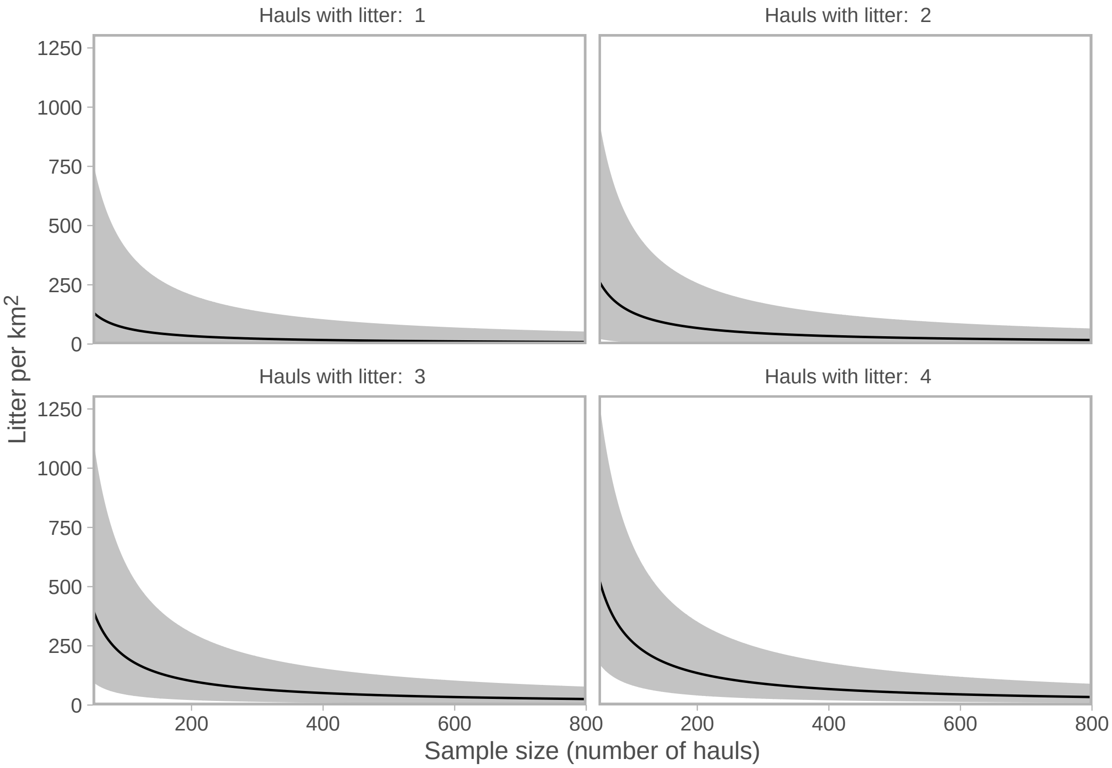

Figure S5: Litter density estimates and 95% CI for varying sample sizes (number of hauls) and number of hauls with litter per sample size using the Agresti-Coull method (note a haul with litter can only contain one litter object in this hypothetical example). Haul area: 0.000148 km^2. The solid line depicts the mean and the ribbon covers the 95% confidence interval.
