# Supplementary material for: Evaluating the potential of underwater television to contribute to marine litter assessments alongside bottom trawling
Source: PLoS One. 2025 Jun 27;20(6):e0324900. doi: 10.1371/journal.pone.0324900 (PMC12204539; doi:10.1371/journal.pone.0324900)
Supplement: S1 Table — AIC and ΔAIC (AIC for the model relative to the model with the lowest AIC) for all spatial and spatiotemporal GLLMs fitted to litter density data. In model 1, we use a spatial random field for the binomial and Gamma components of the delta-model, in model 2, we replace the spatial random field with a spatiotemporal AR1 random field, and in model 3 we use a spatial random field for the binomial model and a spatiotemporal AR1 random field for the Gamma model. (PDF) [file pone.0324900.s006.pdf]

Table S1: AIC and ΔAIC (AIC for the model relative to the model with the lowest AIC) for all spatial and spatiotemporal GLLMs fitted to litter density data. In model 1, we use a spatial random field for the binomial and Gamma components of the delta-model, in model 2, we replace the spatial random field with a spatiotemporal AR1 random field, and in model 3 we use a spatial random field for the binomial model and a spatiotemporal AR1 random field for the Gamma model.

**Model**	**binomial**	**Gamma**	**AIC**	**ΔAIC**
1	Spatial	Spatial	4287	8
2	Spatiotemporal	Spatiotemporal	4279	0
3	Spatial	Spatiotemporal	4281	2
